# Supplementary material for: Are carbon emissions trading and green financial instruments synergistic? -Comprehensive quantitative research based on content analysis
Source: PLoS One. 2024 Mar 7;19(3):e0298601. doi: 10.1371/journal.pone.0298601 (PMC10919601; doi:10.1371/journal.pone.0298601)
Supplement: S1 Data — (ZIP) [file pone.0298601.s001.zip › Data/地级市Z_F工作报告.zip!/╔╜╢1⁄2/╟α╡║╩╨/└┤╫╘╣1⁄2╓┌║┼í╛┬φ┐╦╩2╛▌═°í┐_2017.docx]

各位代表：

　　现在，我代表市人民政府向大会报告工作，请予审议，并请各位政协委员和其他列席人员提出意见。

　　一、过去五年工作回顾

　　过去五年，在以习近平同志为核心的党中央坚强领导下，按照中央和省、市委的部署要求，我们深入学习贯彻党的十八大和十八届三中、四中、五中、六中全会精神，深入学习贯彻习近平总书记系列重要讲话精神和党中央治国理政新理念新思想新战略，深入学习贯彻习近平总书记视察山东重要讲话、重要批示精神，坚持世界眼光、国际标准，发挥本土优势，积极寻标对标达标夺标创标，努力加速提升创新增效落实，完成了市第十五届人大一次会议确定的主要任务，谱写了宜居幸福现代化国际城市建设的新篇章。

　　五年来，全市生产总值年均增长8.9%，2016年达到10011亿元；一般公共预算收入年均增长14.2%，达到1100亿元；规模以上工业总产值年均增长10.4%，达到18280亿元；固定资产投资年均增长17.4%，达到7455亿元；实际到账外资年均增长14%，达到70亿美元；社会消费品零售总额年均增长12.3%，达到4105亿元；城乡居民人均可支配收入分别年均增长9.4%、10.4%，达到43598元、17969元。

　　产业结构持续升级。聚焦“转调创”、发力“蓝高新”，加快实施“腾笼换鸟、凤凰涅槃”行动，打造青岛经济升级版。持续开展“调稳抓”现场推进活动和“三个一千”活动，一汽大众华东生产基地、东方影都、慧与全球大数据基地等26个百亿元以上项目加快建设，北汽新能源汽车等一批重大项目竣工投产。111户老城区企业实现环保搬迁。持续推进“四个十”工程，服务业增加值占比达54.7%，提高6.9个百分点。十条千亿级产业链占规模以上工业总产值比重超过75%，战略性新兴产业比重达20%。互联网工业异军突起，智能机器人、三维打印、石墨烯等新兴产业蓬勃发展。软件业务收入跨上千亿元台阶。海洋经济占生产总值比重达25.1%。农业综合效益显著提升，农产品出口额位居全国城市首位。

　　空间布局不断优化。实施“全域统筹、三城联动、轴带展开、生态间隔、组团发展”的城市空间发展战略，海湾型大都市格局初步显现，成为国家沿海重要中心城市。“一谷两区”等重点区域迅速崛起，西海岸新区生产总值稳居国家级新区前三强，高新区纳入国家自主创新示范区，蓝谷列入国家“十三五”规划。胶东国际机场开工建设。胶东临空经济示范区获国家批复。机场旅客吞吐量达2050.5万人次。接待游客总人数达8081万人次。港口货物和集装箱吞吐量分别达到5.15亿吨和1805万标箱。国际邮轮港、铁路青岛北站建成启用。青荣城际铁路建成通车，济青高铁、青连铁路、青平城际铁路加快推进。完成城市轨道交通线网规划，在建305公里，省内首条地铁建成通车。新型城镇化扎实推进，小城市培育和特色镇创建初见成效，常住人口城镇化率达71.5%。四市全部进入全国百强县（市）行列。

　　发展动能加快转换。坚持把发展基点放在创新上，搭建平台、集聚资源、优化生态，取得显著成效。海洋科学与技术国家实验室、国家深海基地投入使用。高速列车技术创新中心成为全国首个国家技术创新中心。中科院在青形成“两所八基地一中心一园一城”的发展格局。山东大学、复旦大学等29所重点院校相继落户。中船重工等16家国字号科研产业基地建成运营。国家级创业孵化载体达96家。高新技术企业达1348家，增长2.3倍。发明专利授权量、PCT国际专利申请量、技术合同交易额分别增长4.8倍、5倍、4倍。实施百万人才集聚行动，住青院士26人、聘任院士23人，泰山学者115人，人才总量169万人。

　　发展活力更加强劲。坚持以改革破难题、以开放促发展，改革红利不断释放，开放型经济再创新优势。实施了经济、社会、文化、生态等领域600多项改革任务。完成新一轮政府机构改革。推进简政放权、放管结合、优化服务改革，行政审批事项精简57%。深化商事制度改革，市场主体总量突破100万户。市直企业经济效益在全国城市中居第4位。民营经济税收占比达44.2%。财富管理金融综合改革试验区建设成效显著，实现境内直接融资452亿元，金融业增加值占生产总值比重达6.7%。获批国家国内贸易流通体制改革发展综合试点城市。成为国家级旅游业改革创新先行区和全国旅游标准化示范城市。农村集体产权制度改革全面推行，农村土地“三权”分置改革顺利推进，确权登记颁证任务基本完成。

　　对外开放实现新突破。实施国际城市战略，成为国家“一带一路”战略节点支点城市，对沿线国家投资额、出口额占全市比重分别达27.7%、53.1%。国际贸易总额突破1000亿美元。实际利用外资稳居副省级城市前列。“走出去”企业达1400多家，增长近2倍。新开通4条洲际直航航线，国际航线达到19条。董家口港获批国家一类开放口岸。保税港区汽车口岸成为全国第五大整车进口口岸。多式联运海关监管中心建成运营。新增14个友城、57个国际经济合作伙伴城市，成为中美、中英地方经贸合作重点示范城市。高水平承办APEC贸易部长会议、C20民间组织会议、国际教育信息化大会等一批重要国际活动。累计利用国内资金6822亿元。对口支援工作扎实推进。

　　生态环境明显改善。强化城市生态空间管控，推动美丽青岛建设，绿色发展水平不断提升。加强胶州湾综合整治，胶州湾优良水质面积比例由52.9%提高到72.1%。成为国家级海洋生态文明建设示范区。大沽河成为统筹城乡、贯穿南北的生态走廊。李村河等20条重点河流水质持续改善。城市污水集中处理率达98.6%。全面完成国家、省下达的节能减排降碳任务。清洁能源供热面积超过3500万平方米。空气优良天数达到299天。新增造林63.7万亩，林木绿化率为40%，获批国家森林城市。成功举办2014世界园艺博览会。实施农村环境连片整治，城乡环卫一体化实现全覆盖。

　　民生质量显著提高。坚持保基本保公共保普惠保均等，大力实施惠民工程，城乡居民获得感进一步增强。全市财政用于民生的投入达3915亿元，年均增长19.4%。建立城乡一体的就业创业政策体系，成为全国创业先进城市、国家新型职业农民培训示范市。学前教育普及水平、义务教育校际均衡水平位居副省级城市第一。建成齐鲁医院青岛院区一期、三医、妇儿医院、和睦家医院等一批重点医疗项目。医药卫生体制改革不断深化，居民医保个人负担率明显下降。长期护理保险制度荣获政府创新最高奖。千名老人养老床位从13张增加到37张，政府为困难老人购买居家养老服务实现全覆盖。人均期望寿命提高到80.9岁。医养结合“青岛模式”全国推广。城乡低保标准分别提高54.8%、116.6%。市定标准10万贫困人口全部脱贫。筹建保障性住房7.2万套。完成棚户区改造13.4万户。累计建成新型农村社区269个，35.4万农民搬进新居。持续开展重点领域专项治理，安全生产形势平稳，食品药品安全整治深入推进，交通拥堵治理成效显现，市容市貌整洁有序，社会治安环境持续优化。

　　社会文明全面进步。注重物质文明与精神文明协调发展，推进文化青岛建设，城市文化软实力再上新台阶。积极培育和践行社会主义核心价值观，群众性精神文明建设实现常态化。蝉联全国文明城市称号。成为首批国家公共文化服务体系示范区。31件作品获“五个一工程”等国家奖项。首次荣获文华大奖、鲁迅文学奖。历史文化名城保护全面加强。全民健身蓬勃开展。世界休闲体育大会成功举办。青岛籍运动员在国内外大型赛事上取得优异成绩。军民融合创新示范区建设全面推进，军政军民关系更加巩固，实现全国双拥模范城“八连冠”。成为全国首批法治城市创建活动先进市。法律援助办案量保持全省第一。民族宗教、外事、侨务、对台、史志、统计、仲裁、档案工作不断加强，人防、气象、防震减灾、应急工作扎实推进，妇女、儿童、老龄、慈善、残疾人和红十字事业健康发展。

　　政府建设扎实推进。坚持依法行政、从严治政，全面履行政府职能，政府公信力和执行力进一步提升。认真落实向人大报告、向政协通报制度。坚决执行人大及其常委会的决议、决定，自觉接受人大法律监督、工作监督和政协民主监督。向市人大常委会提交地方性法规草案23件，制定政府规章38件，办复市人大代表建议2747件、市政协提案3368件。严格落实重大行政决策制度。完善信息公开制度，建成网上服务大厅和政府数据开放网站。持续开展向市民报告、听市民意见、请市民评议活动。政府部门网络在线问政形成常态。强化政府廉政建设主体责任和领导干部“一岗双责”，全面从严治党主体责任得到有效落实。推进行政监察和审计监督全覆盖，深化权力集中领域的改革，廉政风险得到有效防控。从严问责失职行为，行政问责740起、纪律处分127人。坚决查处“四风”、严厉惩治腐败，纪检监察机关累计立查案件5351件，党纪政纪处分5150人。扎实开展党的群众路线教育实践活动、“三严三实”专题教育、“两学一做”学习教育，认真落实中央八项规定精神，不断强化“庸懒散慢拖瞒”治理，营造了尚实干、勇作为、敢担当的良好政治生态。

　　各位代表！回首五年发展历程，成绩来之不易。这些成就，归功于党中央国务院、省委省政府和市委的坚强领导，归功于市历届领导班子的励精图治，归功于市人大、市政协和社会各界的监督支持，归功于全市人民的拼搏奉献。在此，我代表市人民政府，向全市人民，向全体人大代表、政协委员，向各民主党派、各人民团体、社会各界人士和离退休老同志，向中央、省驻青单位，向驻青部队和武警官兵，向所有关心支持青岛发展的海内外朋友们，表示衷心的感谢和崇高的敬意!

　　在肯定成绩的同时，我们也清醒地看到面临的挑战和存在的不足，主要是：经济增长内生动力仍需增强，一些企业生产经营存在困难。经济发展中仍然存在一些结构性矛盾，高端产业引领作用不够强，新旧发展动能转换任务艰巨。自主创新和成果转化能力有待提升，人才总量和结构还不适应创新发展的需要。全域统筹发展存在不少短板，城乡公共资源布局不够均衡。水资源总量不足，农村面源污染问题亟需解决，环境治理水平仍需提高。公共服务有效供给不足，民生工作还有一定差距。服务型政府建设任重道远，有的部门和干部缺乏改革勇气和担当精神，依法行政意识和水平有待提高，一些领域腐败问题时有发生。我们将强化忧患意识，正视问题、直面挑战，努力加以解决。

　　二、今后五年的目标任务

　　各位代表！市第十二次党代会站在新的历史起点，绘就了青岛未来发展的宏伟蓝图。按照党代会的战略部署，今后五年政府工作总的要求是：紧密团结在以习近平同志为核心的党中央周围，高举中国特色社会主义伟大旗帜，坚持以马克思列宁主义、毛泽东思想、邓小平理论、“三个代表”重要思想和科学发展观为指导，深入贯彻习近平总书记系列重要讲话精神和党中央治国理政新理念新思想新战略，全面贯彻党的十八大和十八届三中、四中、五中、六中全会精神，认真学习贯彻党的十九大精神，统筹推进“五位一体”总体布局和协调推进“四个全面”战略布局，认真践行以人民为中心的发展思想，坚持稳中求进工作总基调，贯彻落实新发展理念，落实省委“一个定位、三个提升”要求，以推进供给侧结构性改革为主线，以建设“三中心一基地”为主攻方向，不断提高发展质量和效益，提升城市品质和能级，增强集聚效应和辐射能力，争取进入国家中心城市行列，加快建设宜居幸福创新型国际城市。

　　今后五年经济社会发展主要目标是：保持经济稳中有进、进中向好，生产总值年均增长7.5%左右，发展质量效益进一步提高，城乡居民人均收入提前实现翻一番目标，城市国际品质、竞争力和宜居水平明显提升，市民生活更加舒适便捷，全面建成更高水平更高质量小康社会。

　　（一）坚持创新发展，实现发展动能新突破。深入实施创新驱动发展战略，加快实现新旧发展动能转换，建设具有重要影响力的创新之城、创业之都、创客之岛。努力推进实体经济与时俱进发展壮大，产业迈向中高端水平，战略性新兴产业拉动作用明显增强，新经济初具规模，“青岛制造”的规模、品质和地位明显提升，成为全国服务经济先行区和示范区，进入世界海洋经济发展先进城市行列。建设为企业、居民和游客提供最大便利，宜居宜业宜游的智慧城市。全社会研发投入占比达到3.2%以上。高新技术产业产值占规模以上工业产值比重46%以上。服务业增加值占比57%左右。

　　（二）坚持协调发展，构建全域统筹新格局。传承全域统筹发展规划，深化“三带一轴、三湾三城、组团式”的城市空间布局，建设国际湾区都会。区域协同、城乡一体、物质文明精神文明协调、军民融合发展水平显著提高。建成胶东国际机场、济青高铁、青连铁路、潍莱高铁。每年至少开通一条地铁，轨道交通营运里程260公里以上。建设10个现代化次中心城市，打造35个特色镇（街道）、300个特色城乡社区（村）。常住人口城镇化率提高到74%以上。

　　（三）坚持绿色发展，推动生态环境新提升。严格“七线”管控，保护好“红瓦绿树、碧海蓝天”的城市风貌。打好大气、水、土壤污染防治三大战役，大气PM2.5年均浓度下降20%以上，海洋生态环境持续改善，彻底消除河道、水库、塘坝黑臭水体，土壤环境质量安全稳定。基本实现城乡生活垃圾分类收集全覆盖。清洁能源消费比重持续提高。建立领导干部自然资源资产离任审计和生态环境损害责任终身追究制度。生产生活方式更加绿色，城乡人居环境更加美好。

　　（四）坚持开放发展，推进对外开放新跨越。驰而不息抓开放促发展，深度融入“一带一路”战略，加快推进国际城市建设。进一步打造公开透明、法治规范、充分便捷、与国际惯例相衔接的营商环境，国际贸易、利用外资和对外投资总额保持同类城市领先水平。积极打造国际化的教育、医疗、养老服务体系，外籍常住人口、留学生、入境旅游人数大幅增长。建成东北亚国际航运枢纽、国家区域性航空枢纽、国家沿海高铁枢纽，打造欧亚大通道多式联运中心和转口贸易枢纽。大力引进国内外的资金、人才、技术和装备，支持有实力的企业有序走出去，发展一批总部在青岛的跨国公司，建成一批境外产业园区，构筑国际城市的坚实支撑。

　　（五）坚持共享发展，促进民生福祉新改善。坚持以人民为中心的发展思想，努力使城乡居民劳有应得、学有优教、病有良医、老有颐养、住有宜居、贫有力助。就业、教育、文化、医疗、养老、住房、社保、交通、治安等公共服务和保障体系进一步健全，基本公共服务质量和均等化水平持续提升，工作生活的便利性明显提高，参与感、获得感、幸福感不断增强。食品安全、清洁取暖等群众关注的热点难点问题得到有效解决。城镇棚户区和农村危房改造任务全面完成。实现农村、城镇贫困人口稳定脱贫。

　　各位代表！当前，青岛正站在承前启后、继往开来的新起点上。国家赋予青岛沿海重要中心城市的定位，省委省政府要求青岛充分发挥龙头带动的作用，全市人民期待青岛有更加美好的未来。市第十二次党代会为今后发展提出了新目标、指明了新方向。面对宜居幸福创新型国际城市建设的新使命，需要我们提升定位、拓宽视野，开拓创新、埋头苦干，作出一流业绩，创造新的辉煌。面对城市间竞争日趋激烈的新态势，需要我们主动作为、奋发进取，敢于担当、勇于突破，增创发展优势，率先走在前列。面对人民群众对美好生活的新期待，需要我们牢记宗旨、一心为民，脚踏实地、多办实事，抓好民生建设，增进民生福祉。我们坚信，有党中央国务院、省委省政府和市委的坚强领导，有全市人民的团结拼搏，我们一定会不辱使命、不负重托，圆满实现奋斗目标，交出经得起历史和实践检验、人民满意的答卷！

　　三、2017年主要工作安排

　　完成今后五年的目标任务，今年至关重要。我们一定要积极进取、真抓实干，主动适应把握引领经济发展新常态，全面做好稳增长、促改革、调结构、惠民生、防风险各项工作，实现经济平稳健康发展和社会和谐稳定。主要预期目标是：全市生产总值增长8%左右，一般公共预算收入增长8.5%左右，固定资产投资增长12%左右，社会消费品零售总额增长10.5%左右，国际贸易总额增长5%左右，居民人均可支配收入增长8%左右，城镇登记失业率控制在4%以内，居民消费价格涨幅控制在3%左右，完成年度节能减排降碳任务。重点做好以下工作：

　　（一）深入推进供给侧结构性改革

　　推动“三去一降一补”重点任务取得实质性进展。坚持“一业一策”，分行业制定转型升级方案和政策措施，促进有市场的传统行业转型升级，加快前景好的新兴产业发展壮大，减少无效和低端供给，扩大有效和中高端供给。加快环保搬迁项目建设，促进石化区调整转型，实现老城区污染企业基本关停。鼓励企业兼并重组，推进劣势企业破产清算，加快“僵尸项目”出清。坚持住房的居住属性，合理增加住房有效供应，发展和规范住房租赁市场，整顿房地产交易秩序，努力保持房价稳定。支持企业市场化、法治化债转股，降低企业杠杆率。落实国家税费减免政策，清理规范政府性基金和经营服务性收费，落实进出口环节收费减免政策，努力减轻企业负担。

　　深化农业供给侧结构性改革。推进标准化生产，加强质量安全监管，增加绿色优质农产品供给，打造国家现代农业示范区、国家农产品质量安全示范市。建设30万亩粮油生产功能区和30万亩高效设施农业生产功能区。培育新型农业经营和服务主体，农业规模化经营比重达到65%以上。培育休闲农业、乡村旅游等新产业新业态，促进一二三产业深度融合发展。出台农村土地所有权、承包权、经营权分置办法，完善农村产权交易市场体系。建立农业信贷担保机制。完成1000个村庄集体产权制度改革任务。

　　（二）深入推进国家东部沿海重要的创新中心建设

　　把创新摆在发展全局的核心位置，推进以科技创新为核心的全面创新，大力培育新技术新产业新业态新模式。加快建设虚拟现实、深海与海工装备等支撑产业发展的科技创新中心，超前谋划脑科学、人工智能等面向未来的科技创新中心。推进国家高速列车技术创新中心建设，打造具有全球影响力的高速列车创新高地。推动中科院青岛科教园和在青院所建设发展。支持北京航空航天大学等5所高校新校区建设，推动同济大学等高校在青设立研究生院和研究院，争取中国社会科学院大学、中国康复大学等高校落户。推进西门子青岛研究院等研发机构建设。国家级创业孵化载体达到100家。力争技术合同成交额达到120亿元。加强知识产权保护，开展重点产业专利运营试点。强化企业创新主体地位和主导作用，构建政府积极推动的产学研协同创新机制。深入实施“千帆计划”，高新技术企业超过1500家。加强各领域管理创新，降低运营成本，提升管理效能。鼓励企业开展商业模式创新，促进互联网与传统产业深度融合，发展柔性化设计、个性化定制、供应链管理、制造服务化等新模式，培育平台经济、分享经济、体验经济等新形态。

　　实施人才优先发展战略。完善人才激励保障机制，创新顶尖人才奖励资助、高端人才团队引进支持等政策，加强“高精尖缺”人才的精准引进。允许科研人员和高校教师依法依规适度兼职。推进孵化器、软件园、人才公寓建设，加快人才改革试验区、国际院士港、院士智谷、人才特区、国际人力资源服务产业园发展，完善人才服务中心功能，营造一流人才环境。推动形成鼓励创新、宽容失败的文化氛围。

　　（三）深入推进国家重要的区域服务中心建设

　　推动生产性服务业向科技型、价值链高端延伸。努力集聚更多的服务业企业，充分挖掘其对我市经济发展的支持保障功能，积极推动其对周边地区发挥辐射作用。推进浪潮云计算数据中心、亚马逊联合创新中心等项目建设，支持黄岛争创大数据综合试验区。引进优质金融资源，发展金融新业态，打造面向国际的财富管理中心和金融改革创新试验区。做好政策支持和服务工作，引进新设一批产业投资基金。支持蓝海股权交易中心做大做强。引进专业化平台企业，规范发展大宗商品交易平台。加快港航联合商品贸易场建设，强化国际航运服务中心功能。抓好现代物流创新发展城市试点，推动董家口港区、胶东空港等物流园区建设，加快北方水产品交易和冷链物流基地建设。引进创办一批具有国际影响力的品牌会展项目。积极申办国际海事防务展。发展创意设计、营销策划、咨询评估等商务服务业。推动中铁世界博览城、红岛国际会展中心等100个重点项目建设，争取全年服务业投资450亿元以上。

　　推动生活性服务业向智慧型、高品质转变。适应人民群众不断变化和日益提高的消费需求，鼓励和支持传统服务业改造升级、新兴服务业加快发展。发展文化、旅游、体育、健康、养老等幸福产业。试点建设智慧商圈和智慧商店。建成1-2处10万平方米以上的区域性品牌化二手车市场。高标准规划红岛商贸区，开工建设红岛国际农产品交易中心。推进国家级旅游业改革创新先行区建设，适应大众旅游井喷和高端休闲度假快速增长的新趋势，大力培育新业态，打造国际化旅游目的地城市。加快旅游资源整合与创新发展，培育大型现代旅游企业。

　　推动公共服务业向便利型、高效率提升。统筹规划公共服务业发展，加强公共服务设施建设，在公共管理、社会保障、教育培训等领域开辟服务新空间，努力增加公共产品和公共服务的有效供给。鼓励民间资本参与公共服务业发展。

　　（四）深入推进国际先进的海洋发展中心建设

　　提高海洋科技创新能力。积极融入和服务海洋强国战略，促进海洋科研机构联合创新，抢占海洋科技创新制高点。支持海洋科学与技术国家实验室、深海基地、深海钻探基地、海洋设备质检中心等国字号创新平台提升发展水平。推进全国首个国家海洋基因库建设。做大做强国家海洋技术转移中心，加速海洋科技成果转化。

　　大力发展海洋经济。统筹海洋深度开发和海岸高效利用，加快深远海开发战略保障基地建设，拓展蓝色发展空间。培育海洋信息服务、海洋文化体验、海洋休闲旅游等新型业态，提升海洋生物、深海探测、海洋腐蚀防护等产业水平。转变现代渔业发展方式，发展现代化海洋牧场，打造“蓝色粮仓”。抓好总投资1200亿元的140个重点项目，促进蓝色经济跨越发展。

　　（五）深入推进具有国际竞争力的先进制造业基地建设

　　加快制造业向中高端迈进。发展智能制造、精品制造、绿色制造、服务型制造，建设“中国制造2025”试点示范城市。坚持转型发展，强化企业技术改造，建设4个省级制造业创新中心，培育60家市级以上企业技术中心和工业设计中心，滚动推进300个以上工业转型升级项目，实施1500项企业技术创新重点项目。坚持融合发展，推广海尔、海信等企业经验，培育80个互联网工业试点示范项目，争创5个省级以上智能制造和服务型制造试点示范项目。坚持集群发展，打造轨道交通装备、汽车、航空航天等高端制造高地。坚持超前发展，培育新材料、生物、新能源等战略性新兴产业，发展传感器、石墨烯、智能机器人等新兴制造。

　　统筹推进标准、质量、品牌建设。办好第一届国际标准化论坛，建设标准国际化研究中心。开展“增品种、提品质、创品牌”专项行动，倡导“工匠精神”，加快迈入质量时代。鼓励企业申报中国质量奖、争创中国驰名商标，培育一批国际知名自主品牌。推动“老字号”振兴发展，打造更多“百年老店”。

　　（六）着力提高发展质量和效益

　　优化结构提高产业增加值。在推动经济总量规模继续攀升的基础上，更加注重质量和效益，努力创造更多财富。突出高端高质高效方向，着眼服务业做优做活、工业做强做大、农业做精做特，使各产业在转调创中不断增体量、扩能量、提质量，加快向价值链高端跃升。

　　增强企业盈利能力。引导企业加快产品升级换代，提高竞争力和附加值，开拓市场、提质增效。鼓励企业创新机制、优化管理，瘦身健体、挖潜增效。加强银企对接与产融合作，引导企业利用境内外低成本资金等多元融资、降本增效。

　　提高城乡居民收入。确保居民收入增长与经济增长同步。发布企业工资指导线和人力资源市场工资指导价位，引导企业职工工资合理增长。继续提高退休人员基本养老金，确保按时足额发放。通过支持农民转移就业、农业产业化等综合性措施，保持农民增收势头。

　　促进财政收入稳步增长。优化财源建设政策措施,鼓励区市财政收入增量提质，提高税收收入占比。加强财政预算管理，确保财政资金和公共资源的使用依法合规、合理高效。

　　（七）大力培育和发展壮大市场主体

　　促进市场主体加速成长。坚持抓主体促发展，打造更有活力的创业创新生态，提高经济活跃度。出台培育发展市场主体意见，新增市场主体10万户以上。通过深入挖潜催生一批市场主体，放宽准入、降低门槛，鼓励大众创业、万众创新，扶持个体工商户和小微企业发展壮大。通过扶弱促强壮大一批市场主体，支持个转企、小升规、规改股、股上市，力争新增境内外上市公司5家、新三板挂牌公司25家、蓝海挂牌企业150家。通过市场并购重组一批市场主体，在竞争合作中扩能升级。通过“走出去”拓展一批市场主体，积极参与国际分工，培育更多全国性企业和跨国公司。

　　加大对企业扶持力度。实施便民助企服务清单管理，推进“多证合一、一照一码”和企业登记全程电子化改革。深化大企业直通车和中小企业公共服务体系建设，积极帮助企业解决土地、融资、人才、创新和市场推广等发展难题。发挥各类商会、协会在联系政府、企业和市场中的桥梁纽带作用。优化政策和环境，促进民营经济发展壮大。严厉打击侵犯知识产权等违法行为，维护企业合法权益。

　　尊重企业和各类市场主体，尊重企业家和各类创业者，关心爱护产业工人。激发和保护企业家精神，重视企业家队伍建设，积极为企业家搭建干事创业舞台。多层次培养工程师、技师等实用型人才。重视保护职工合法权益。努力构建既“亲”又“清”的新型政商关系。

　　（八）积极落实区域发展战略

　　推进重点功能区高端突破。推动西海岸新区加快转型升级，提升功能定位，打造海洋强国战略新支点、全省对外开放桥头堡、全市创新发展排头兵。加快蓝谷海洋经济发展示范区建设，做强蓝谷省级区域创新中心，打造世界级海洋创新高地。提升高新区创新发展水平，培育壮大软件信息、医药医疗、智能制造与新材料等六大主导产业，在山东半岛国家自主创新示范区建设中发挥主力军作用。加快胶东临空经济示范区建设，打造区域性航空枢纽、高端临空产业基地、对外开放引领区和现代化生态智慧空港城。推动开发区和各产业园区二次创业，打造改革开放新高地、创新发展新龙头。推进青岛开发区国家智能化工业园区试点示范工作。支持保税港区申建自贸试验区。加快出口加工区向综合保税区转型升级。推动中德生态园打造具有可持续发展示范意义的生态园区。加快建设国际邮轮港城，打造国际化的邮轮旅游发展实验区和金融贸易创新示范区。争取军民融合创新示范区早日获得国家批复。

　　促进区域城乡协调发展。统筹推进老城区复兴发展、新区高品质建设和三城互联互通，缩小南北差距，促进东西均衡。加快市南时尚中心区和青岛湾周边老城区、市北中央商务区和新都心、李沧楼山片区和院士双创中心、崂山湾国际生态健康城和金家岭金融区、城阳动车小镇等区域建设，有序推进老工业区改造，增强辐射带动能力。支持即墨汽车产业城、胶州经济技术开发区、平度新河化工产业基地、莱西新能源汽车基地等建设发展，提升县域经济能级。加快美丽乡村标准化建设，重点建设10个集聚类示范社区、100个美丽乡村示范村和1000个达标村。实施农村“七改”工程，改善人居环境。改造农村旱厕50万座以上，基本完成农村无害化卫生厕所改造任务。深化户籍制度改革，引导农业转移人口有序市民化。推进县域基本公共服务均等化，加强乡镇政府服务能力建设，基本实现标准化农村服务中心建设全覆盖。

　　（九）突出重点项目抓好各类投资

　　开展常态化、精准化招商。立足青岛挖潜，同时眼睛向外，瞄准世界500强、全球行业领军企业、国内500强和民营企业500强，开展“千企招商大走访”活动，大力引进有生命力的优势产业项目、有爆发力的战略新兴产业项目、有带动力的现代服务业项目、有竞争力的特色农业项目、有承载力的城市建设项目。加大政策创新力度，制定更具竞争力的招商引资政策措施。创新招商方式，扎实开展主题招商、委托招商、中介招商和以商招商。

　　加快重点项目建设。抓好总投资7543亿元的200个市级重点建设项目，新开工110个左右，竣工60个以上，完成投资1600亿元以上。加快一汽大众华东生产基地等项目建设，推进空客直升机、清华启迪科技城等项目尽早开工。强化土地、资金等要素保障，抓好项目调度和督查考核，确保重点项目早开工、早竣工、早达产。

　　促进民间投资稳定增长。落实促进民间投资政策措施，激发民间投资活力。强化项目联审联评机制，筛选和推介一批有较好现金流、有稳定回报预期的项目，抓好总投资1540亿元的28个PPP项目，推动PPP合作模式覆盖更多领域。

　　（十）采取切实有效举措扩大消费

　　深入实施扩消费行动。加快培育现代消费经济增长驱动体系，制定服务领域扩大消费实施方案，推进消费“十、三、五”行动计划，争创全国沿海地区国际消费中心城市。积极开拓医养结合、健康管理、康复护理等新服务。扩大数字家庭、在线教育等信息消费。实施扩进口与促消费相结合的政策机制，扩大国外安全优质消费品进口。

　　提高消费便利化水平。大力发展电子商务，推进快递示范城市建设，鼓励电商、快递进社区进农村，打通物流最后一公里。完善现代商贸流通体系，优化商贸均衡布局，加强商贸设施建设，积极引进新业态，适应时代特点和要求发展批发零售业，推进实体店和网络购销融合发展。建设一批退税商场、免税店，引进一批国际品牌。推动“菜篮子”工程惠民便利化、信息化、标准化，创建星级农贸市场40处以上。促进住宿餐饮业转型升级。积极培育社区托幼、家政、保健等便民服务业。加强市场秩序整治，不断优化消费环境。

　　（十一）全方位、深层次推进对外开放

　　深度融入“一带一路”战略。发挥“一带一路”战略节点支点城市功能，加强与“一带一路”沿线国家（地区）的产业和金融等领域合作。复制推广上海等自贸试验区经验，培育参与国际合作竞争新优势。推动东亚海洋合作平台建设，扩大黄岛论坛国际影响力。推进欧亚经贸合作产业园建设。加快布局“一带一路”自贸驿站。

　　加快外经贸动能转换。实施“优质优价优进优出”外贸发展战略，提升自主品牌、高附加值产品出口比重，鼓励先进技术、关键设备和零部件进口。加快跨境电商综合试验区建设，打造邮政跨境电商产业园等一批重点园区，建立一批“海外仓”和境外展示中心。推进中国服务外包示范城市建设。推行国际贸易“单一窗口”，深化关检合作“三个一”，压缩货物通关时间。推行“准入前国民待遇加负面清单”投资管理模式，依法依规放宽外商投资市场准入，加大境外招商引资工作力度，确保实际利用外资稳定增长。积极引进国内外一流企业在青设立地区总部、研发中心、培训基地等辐射全国的功能性机构。拓展境外青岛工商中心布局。完善服务体系和风险防范机制，支持企业积极稳妥地到境外建立原材料基地和生产基地、设立营销中心和研发中心。

　　构建全方位对外开放格局。深入推进国际城市战略，不断深化细化重点行业和领域的对外开放工作。加强国际文化交流，鼓励国内外著名文化创意、制作、经纪、营销机构与本土文化企业合资合作。积极开展中外合作办学。推进科技领域国际合作。鼓励引进国内外高端优质医疗资源。加强城市宣传推介和旅游营销，提升城市知名度和影响力。深化与国际友好城市、友好合作关系城市和经济合作伙伴城市的交流合作。对接国家区域发展战略，扩大与沿黄流域、沿海区域合作，强化与山东半岛城市群和周边地区的联动发展。

　　（十二）积极深化重点领域改革

　　争取更多国家、省改革试点任务，能自主推进的努力先行先试。继续精简下放行政审批事项，建立全市统一的网上审批平台，实施政务服务事项“同城通办”，加快营造审批事项最少、办事效率最高、服务质量最优的环境氛围，构建精简高效的政务生态。推进市场准入负面清单建设。推动监管方式改革创新，实现“双随机一公开”监管全覆盖。加快构建全市统一的公共资源交易平台。深化区市综合行政执法改革。完善旅游综合执法体制机制。取消公立医院、科研院所行政级别，推进法人治理结构建设。推动党政机关、事业单位与所办企业脱钩，实施经营性国有资产统一监管改革。加快行业协会商会与行政机关脱钩改革。鼓励非公有制企业参与国有企业改革。推进市以下财政事权和支出责任划分改革。扩大政府购买服务范围。深化价格机制改革，落实公平竞争审查制度。加快国家新型城镇化综合试点步伐，深化平度国家中小城市综合改革试点。推进镇（街道）行政管理体制改革。开展供销社综合改革。

　　推进金融改革创新。加快财富管理金融综合改革试验区建设，推动青岛财富产品交易中心建设，争取在金融机构引入、人民币跨境投融资、财富管理综合监管等方面取得更多成果。加强金融风险排查和监测预警，强化企业资金链风险防控和新型金融业态监管，推进互联网金融、交易场所等领域风险专项整治，完善金融突发事件应急处置机制，筑牢金融风险“防火墙”。

　　（十三）大力推进城市建设管理运行精细化

　　强化规划引领作用。树立“明智规划”理念，加快建立多规合一、覆盖全域的空间管控体系。编制完成《2049远景发展战略》。加强次中心城市规划编制引导。编制海域资源利用总体规划，加强海岸线规划管理。推进城市设计试点，开展“生态修复”和“城市修补”，提升城市功能品质。

　　推进精品化建设。推动胶东国际机场和董家口疏港铁路等重点铁路工程建设，加快6条地铁线建设，争取2号线东段开通试运营。开辟调整公交线路20条，创建公交都市。推进贫困村、偏远村通公交（客车）。抓好海绵城市、地下综合管廊建设试点，建成地下综合管廊30公里、海绵城市30平方公里。续建、新建、改建城市道路12条，推进6条未贯通道路建设。加强信息基础设施建设，加快城乡宽带网络提速，提高重点公共区域免费WiFi覆盖面,构建集城市建筑云、智慧城管、智慧养老、智慧社区等于一体的服务体系。

　　加强精细化管理。坚持长效化、信息化、高效化、法治化，创新管理方式，提高城乡管理水平。出台一批城市管理行业标准。加大城乡环境一体化综合整治力度。加强综合行政执法，全年拆除违法建设不少于450万平方米。实施交通秩序综合治理，加快城乡智能交通系统建设，构建智能停车一体化平台和停车诱导系统，推行道路停车泊位统一管理，新建公共停车泊位7500个以上。

　　增强运行保障能力。加快水源工程建设，提高引江、引黄输水能力，推进白沙河等5座主要水厂水质深度处理改造项目，开工建设日处理能力5万吨的海水淡化矿化项目，再生水利用率达38%以上。完成麦岛污水处理厂升级改造，建成张村河水质净化厂。实施“五个三千”工程，推进“世界一流城市配电网”试点。建设电梯安全应急和监控中心。新建天然气管网180公里，新增天然气用户8万户。优化环卫设施布局，城镇生活垃圾无害化处理率达到100%。

　　（十四）着力保障和改善民生

　　人民对美好生活的向往，就是我们的奋斗目标。必须持之以恒为群众办实事、解难事，让人民生活更有保障、更有尊严、更加幸福。重点办好10件33项市办实事。

　　大力促进就业创业。落实就业优先战略，做好高校毕业生和城镇困难人员、残疾人就业工作，确保城镇零就业家庭“动态消零”，实现城镇新增就业30万人。实施大众创业工程，支持创业投融资平台发展，政策性扶持创业1.5万人。完善覆盖城乡的劳动者技能培训制度，技能培训帮扶1万人，新型职业农民技能证书培训1.5万人。完善协调机制，构建和谐劳动关系。健全农民工工资支付督促检查和治理机制。

　　办好公平优质教育。启动省级教育综合改革试验区建设。新建、改扩建100所幼儿园，全市公办和普惠性幼儿园比例达到80%以上。推动义务教育优质均衡发展，新建、改扩建中小学75所，改造农村义务教育薄弱学校100所，新建中小学标准化食堂80所。开展城区优质学校和农村中小学校结对帮扶工作。统一城乡义务教育学生“两免一补”政策。加强农村教师队伍建设，落实乡村教师支持计划。全市95%的中小学校建成智慧校园。优化职业学校和专业布局，打造品牌职业院校。支持驻青高校积极实施“双一流”建设。推进家庭教育普及工程。健全终身教育体系。提高特殊教育水平。扶持规范民办教育发展。

　　深化健康青岛建设。落实健康中国规划,努力让人民群众公平可及地享有全方位、全周期的健康服务。加快市公共卫生中心、市民健康中心、北部医疗中心等重点医疗项目建设。启动医疗健康数据中心建设，发展智慧医疗、精准医疗。深化医药卫生体制改革，推行医药分开，巩固基本药物制度，推进医联体建设。加快推进国家中医药综合改革试验区和省级健康服务业创新发展试验区各项工作。开展健康社区建设。推进分级诊疗制度，推行家庭医生签约服务。加强乡村医生、全科医生队伍建设。完善医疗纠纷调解机制。强化院前急救网络体系建设。加强妇幼健康、孕前优生服务，提高出生人口素质。

　　扎实推进脱贫攻坚。实施脱贫退出与监测动态管理，健全稳定脱贫长效机制。加快贫困镇村基础设施和基本公共服务建设，抓好产业扶贫，深入开展金融扶贫、教育扶贫、健康扶贫等扶贫行动，实现省定贫困村和市定经济薄弱村全部脱贫摘帽。启动城镇精准扶贫工作。鼓励引导社会力量参与扶贫。做好对口支援和扶贫协作。

　　织密扎牢民生保障网。推进住房租赁补贴发放和实物保障工作，完成住房保障6000套（户），启动棚户区改造4.8万套（户），实现回迁2.6万户，完成老旧住宅小区综合整治829万平方米。提高社会保险待遇和城乡低保标准。落实全民参保登记计划，推广社保卡就医“一卡通”。实施补充医疗保险制度，健全城乡一体的医疗保险制度。完善长期护理保险制度，发展农村护理保险业务。加强养老机构服务质量监管和护理人员培养，完善激励机制，提高养老院服务质量。健全医养合作机制，将符合条件的养老机构内设医疗卫生机构纳入城乡医保定点范围。建立10处社区失智老人照护示范中心。完善农村社区居家养老服务体系。发展临终关怀事业。完善重度残疾人护理补贴和困难残疾人生活补贴制度，为全市60周岁以下残疾人购买意外伤害保险。完善医疗救助和临时救助制度。支持慈善事业发展。完善农村留守儿童和妇女、老人、残疾人关爱服务体系。

　　（十五）切实抓好环境保护和资源集约高效利用

　　加强生态保护和建设。推进燃煤废气、机动车尾气、工业有机废气和扬尘治理，完成40台燃煤锅炉超低排放改造等治理项目。严格落实河长制、湾长制，加强流域、海域精细化管理，建设胶州湾、西海岸国家级海洋公园，基本完成14处黑臭水体整治。加强18处城市集中饮用水水源地环境保护与整治，实施农村规模化供水提质改造工程。启动土壤环境状况详查，强化工业污染场地土壤环境管理，加大农业面源污染防治力度，加快畜禽养殖废弃物处理和资源化利用。建设青岛北部生态屏障。加强山体保护，做好浮山综合治理。建设绿地200公顷，新增造林2.3万亩。

　　推进资源集约高效利用。实施能源和水资源消耗、建设用地等总量和强度双控行动。开展近零碳排放区示范工程，扩大清洁能源利用，推进重点用能单位节能低碳行动，推广装配式建筑、被动屋等节能技术，实施节能保暖工程200万平方米，更新新能源公交车914辆。提倡全民节约用水，推广节水技术，全面建立居民用水、用气阶梯价格制度。实行最严格的耕地保护制度，划定全市永久基本农田。

　　完善生态文明制度。落实省级生态红线，开展市级生态红线划定工作。实施碳排放权、排污权、水权等有偿使用和交易等制度。健全生态保护补偿制度。建立新改建城市绿地补助机制。推行环境污染第三方评估和治理。着力解决环境突出问题，严厉查处环境违法违规行为。

　　（十六）全面提升城市文化品质

　　提升城市文明程度。大力培育和践行社会主义核心价值观，传承发展中华民族优秀传统文化。广泛开展“中国梦”主题教育实践，推进“四德工程”建设。深化文明城市、文明单位、文明村镇、文明社区、文明校园、文明家庭创建活动，努力营造向上向善的城市文化。开展文明交通等系列宣传活动。推动志愿服务制度化。加强未成年人思想道德建设。推进网络文化建设。深入开展双拥共建，加强国防教育和民兵预备役工作。推动人防建设与经济社会融合发展。推进气象、防震减灾能力建设，创建国家防震减灾示范城市。做好侨务、对台和民族宗教工作。发挥好仲裁、司法职能作用。提升统计、档案、史志工作水平。促进老龄、慈善、残疾人、妇女儿童、青少年事业全面发展。

　　建设文化青岛。实施文化惠民提升工程，加强区市公共文化设施和乡镇基层综合文化服务中心建设，办好市民文化艺术节，开展全民阅读活动。推进国家首批文化消费试点城市建设。推动市文化艺术中心开工、市博物馆改扩建、电影博物馆建设。推进即墨故城、琅琊台、齐长城等遗址保护利用项目规划建设。打造滨海文化长廊，彰显海洋文化特色。发展高端影视，申创“电影之都”，打造东方影都、国际电影交易中心和国际电影艺术技术创新平台。设立文化创意产业发展基金，实施文化创意产业“个十百千万”工程。支持媒体深度融合发展。高水平举办电影金凤凰奖颁奖典礼。加强科学普及。繁荣发展哲学社会科学。支持特色新型高端智库发展，加快中国社会科学院青岛研究院建设。加快市民健身中心等场馆建设，打造一批市民身边的活动中心，建设以笼式足球场为主的运动场100处。推进帆船之都和足球名城建设。做好第24届省运会、第10届省残运会等筹备工作。

　　（十七）不断加强和创新社会治理

　　综合运用经济、行政、法律、科技、文化等手段，推进治理体系和治理能力现代化，构筑全民共建共享的社会治理格局。推动安全生产领域改革发展，加快风险分级管控和隐患排查治理体系建设，加大举报奖励和违法处罚力度，坚决守牢安全生产底线。做好森林防火和防汛抗旱工作。加强城乡公共消防设施和道路交通安全设施建设。坚持源头严防、过程严管、风险严控，完善食品药品安全监管体制，加快食品安全标准同国际标准对接，创建国家食品安全示范城市。食品安全定性定量检测6.5万批次。实施“七五”普法规划，建成市、区（市）规范化公共法律服务中心。落实信访工作责任制，依法及时就地解决群众合理诉求。加强行业性、专业性人民调解组织建设，完善矛盾纠纷多元化化解机制。健全立体化、智能化社会治安防控体系，加强社会治安综合治理基层基础建设，建立“一村一警务助理”制度，依法严厉打击各类刑事犯罪。加强对“村霸”和宗族恶势力的整治。加强风险评估，完善应急体系，增强全民自救互救意识，提升基层单位应急管理和第一响应能力。建成企业信用服务系统，完成国家信用体系建设试点任务，力争进入全国首批信用体系建设示范城市。

　　四、努力建设人民满意政府

　　牢固树立政治意识、大局意识、核心意识、看齐意识，坚决维护以习近平同志为核心的党中央权威，把全面从严治党要求落实到政府工作的各个方面，不忘初心、坚守正道，加快转变政府职能、提高行政效能，更好为人民服务。

　　（一）坚持依法行政。深入贯彻法治政府建设实施纲要和加强法治政府建设的意见，将政府活动全面纳入法治轨道。严格执行市人大及其常委会决议决定，落实重大事项向人大及其常委会报告、向政协通报制度，自觉接受市人大的法律监督和工作监督，主动接受市政协民主监督。做好建议提案办理工作。认真听取各民主党派、工商联、无党派人士和人民团体意见。加强城乡建设与管理、环境保护、历史文化保护等领域政府立法。开展相对集中行政处罚权和相对集中行政许可权试点，加快行政执法监督体制机制改革。推进行政复议规范化建设，做好行政应诉工作。坚持科学民主依法决策，完善公众参与、专家论证、风险评估、合法性审查和集体讨论决定等法定程序，落实政府重大决策出台前向市人大报告制度。加强政务诚信建设，完善政务诚信约束和问责机制，以实际行动取信于民。

　　（二）优化政务服务。推行“互联网+政务服务”，推动网上政务服务向基层延伸，加快公共服务事项网上办理，初步实现政务服务“一号申请、一窗受理、一网通办”。清理各种证明和手续，最大程度方便群众和企业。推进政务信息资源整合共享，建成启用政务大数据和云计算中心。探索政务公开标准化、规范化试点，推进行政决策、执行、管理、服务、结果和重点领域信息公开。加强政府网站建设和管理，强化政策解读，回应社会关切。深化政民互动，加强政务服务热线平台建设和政务舆情回应，发挥“三民”活动、网络在线问政、行风在线等互动平台作用，提高政府感知群众期盼、服务公众需求的能力和水平。

　　（三）推进作风转变。始终把纪律和规矩挺在前面，严守党的政治纪律和政治规矩，严格执行中央八项规定精神，确保政令畅通、令行禁止。巩固放大“两学一做”学习教育成果，大力倡树积极进取的精神、务实创新的精神、担当有为的精神，推动改进作风常态化、长效化。加强政府工作人员培训和知识更新，提升专业能力、创新发展能力和依法办事能力。深入基层调查研究，进一步察实情、出实招、办实事。加强对依法履责的监督，完善督查问责机制，以“钉钉子”精神推进工作落细落小落地落实，问责为官不为，惩治为官乱为，切实做到言必信、行必果。健全正向激励机制和容错纠错机制，旗帜鲜明给干事者鼓劲、为担当者撑腰，让广大干部想干事、敢干事、能干成事。

　　（四）强化廉政建设。扭住党风廉政建设责任制这个龙头，坚持严字当头，树立问题导向，拧紧责任链条，把管党治党政治责任与政府工作责任一起落实。强化公共资金、国企国资、金融和公共资源交易等重点领域监管。加强财政支出管理，努力提高财政绩效，政府一般性支出一律压减5%，确保“三公”经费只减不增。加强审计监督和审计查处问题的整改。坚决整治侵害群众利益的不正之风和突出问题，继续“零容忍”惩治腐败。各级政府工作人员要不断增强政治定力、纪律定力、道德定力、抵腐定力，守住从政为官的底线、不越制度法规的红线、筑牢党内监督的防线，始终做到公正用权、谨慎用权、依法用权，以实际行动树立新一届政府的良好形象。

　　各位代表！新的蓝图鼓舞人心，新的征程催人奋进。让我们更加紧密地团结在以习近平同志为核心的党中央周围，在中共青岛市委的坚强领导下，撸起袖子加油干，勇立潮头敢为先，不断开创宜居幸福创新型国际城市建设新局面，以优异成绩迎接党的十九大和省党代会胜利召开!
